# Cuffless Blood Pressure Monitor for Home and Hospital Use

**DOI:** 10.3390/s25030640

**Published:** 2025-01-22

**Authors:** Toshiyo Tamura, Ming Huang

**Affiliations:** 1 Healthcare Robotics Institute, Future Robotics Organization, Waseda University, Tokyo 169-8050, Japan; 2 Shenzhen Institute of Advanced Technology, Shenzhen 518055, China; alex-mhuang@is.naist.jp; 3 Graduate School of Science and Technology, Nara Institute of Science and Technology, Ikoma 630-0192, Japan

**Keywords:** cuffless sphygmomanometer, photoplethysmography, tonometry, standard, medical approval

## Abstract

Cardiovascular diseases, particularly hypertension, pose a significant threat to global health, often referred to as a “silent killer”. Traditional cuff-based blood pressure monitors have limitations in terms of convenience and continuous monitoring capabilities. As an alternative, cuffless blood pressure monitors offer a promising approach for the detection and prevention of hypertension. Despite their potential, achieving clinical performance standards remains a challenge. This review focuses on the principles of the device, current research and development, and devices that are currently approved as medical devices. Then, we describe measures to meet home and clinical performance requirements. In addition, we provide thoughts on validating the accuracy of devices in the home and hospital setting.

## 1. Introduction

Cardiovascular disease (CVD) is the leading cause of mortality globally, with hypertension emerging as a major risk factor. Over the decades from 1990–2019 the prevalence of hypertension has doubled [1], underscoring the urgent need for early detection and intervention strategies. An estimated 1.28 billion individuals worldwide suffer from hypertension, with two-thirds residing in low- and middle-income countries. According to recent US guidelines, normal blood pressure (normotension) is defined as a systolic blood pressure of 120 mmHg and a diastolic blood pressure of less than 80 mmHg [2]. The Japanese Society of Hypertension, aligning with the US standards, revised its guidelines in 2019 to define normotension as readings below 120/80 mmHg. In many countries, daily blood pressure assessments are conducted through unrestricted methods to account for the phenomena as of white coat hypertension and masked hypertension. In Japan, home blood pressure measurement is recommended, especially over a long period of time.

The traditional cuff-based method, which involves placing a cuff on the upper arm and measuring blood pressure during inflation and deflation, remains the predominant technique for blood pressure measurement. Several cohort studies have indicated that home blood pressure monitoring is more predictive of cardiovascular disease and stroke-related morbidity and mortality than office-based measurements [3]. The use of home blood pressure monitoring in routine risk assessment is also endorsed [4]. However, continuous and long-term monitoring of cuff sphygmomanometers presents challenges due to the necessity of maintaining the measurement site at heart level at rest, which may disrupt daily activities and sleep. Therefore, efforts are being made to reduce the size of blood pressure monitors using cuffs.

In recent years, blood pressure estimation without the need for a cuff, which is less stressful for the user, has been considered. Tonometry, pulse wave propagation time, and pulse contour methods have attracted attention as cuffless blood pressure estimation methods [5,6]. Cuffless blood pressure measurement is characterized by the ability to obtain continuous beat-to-beat systolic and diastolic blood pressure without using a cuff.

Blood pressure measurements for health management are generally taken at fixed times, such as morning and evening measurements. However, the clinical significance of continuous blood pressure monitoring has been increasingly recognized, particularly for assessing the efficacy of antihypertensive medications, detecting sudden blood pressure spikes, and understanding nocturnal blood pressure patterns. In operating theaters and ICUs, blood pressure information is crucial, especially in critical illness scenarios, and obtained by invasive means, such as the direct arterial methods, which involves inserting a catheter into an artery. This approach provides real-time and accurate blood pressure data but carries inherent risks due to its invasive nature.

Consequently, volume compensation methods have been proposed as an alternative method. The volume compensation method involves the application of external pressure via a cuff to compress the arteries, maintaining a constant arterial volume that pulsates in sync with the heartbeat. This process equilibrates the pressure at the measurement site (cuff pressure) with the internal arterial pressure, effectively measuring blood pressure. By detecting the cuff pressure when this equilibrium is maintained, continuous blood pressure readings can be obtained. However, this method also carries a risk of stasis due to the cuff vibrating in time with the pulsation of the fingertip, as the cuff is attached to the fingertip to maintain equilibrium of the intravascular pressure. A method of preventing stasis has been proposed by preparing two cuffs for prolonged measurement alternating their activation and rest phases between two fingers. However, concerns remain regarding the reliability of accurate measurements. Notably, the US Food and Drug Administration (FDA) has approved volume clamp methods such as Finapres (Finapres Medical Systems BV, Enschede, The Netherlands), CNAP (CNSystems, Medizintechknik GmbH, Graz, Austria), and the ClearSight system (Edwards LifeScience Corp., Irvine, CA, USA) based on cuff-based sphygmomanometer regulation. Volume clamp device regulation has also been approved as ISO 81060-3:2022 [7], but unlike the auscultation method, this standard does not endorse the use of cuffless continuous sphygmomanometers for validation purposes. There is an anticipated need for the development of simple, non-invasive sphygmomanometers suitable for nocturnal and emergency settings, and the pursuit of a cuffless method of blood pressure measurement is ongoing.

This paper describes the principles of cuffless sphygmomanometers, the devices available on the market, their clinical evaluation, and the development trend of cuffless sphygmomanometers.

## 2. Principles of Cuffless Sphygmomanometer

Cuffless sphygmomanometers are categorized by two primary methods: applanation tonometry and photoelectric pulse wave analysis.

### 2.1. Applanation Tonometry

**Figure 1:**
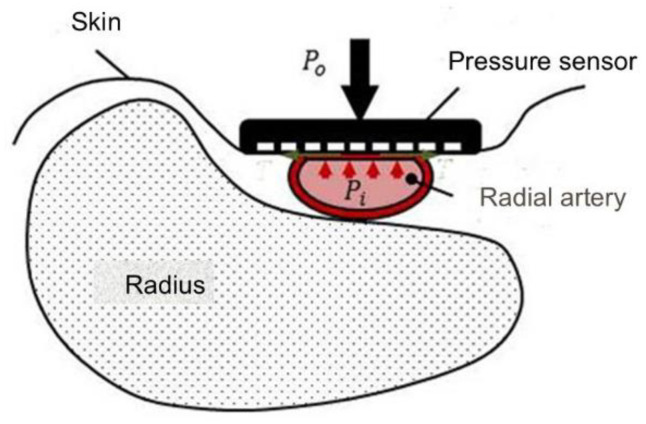
Applanation tonometry.

The applanation tonometric method measures blood pressure per beat by pressing a pressure sensor against an artery close to the body surface, such as the radial artery, as shown in Figure 1. Based on this principle, the pressure sensor must be placed flat against the radial artery in order to accurately measure blood pressure from the radial artery. However, this presents challenges due to the variability in wrist structure among individuals and obstructions posed by the radius and tendons near the radial artery. In addition, it is generally the case that a single sensor is applied to a single point on the radial artery, so it is difficult to know whether the sensor is optimally applied. It is recommended for use under medical supervision [8]. To address these challenges, a method of arranging multiple pressure sensors, or two rows of sensors, and a mechanism that automatically adjusts the optimum sensor angle to suit the individual based on the pressure information obtained from each sensor has been developed. This innovation enables continuous blood pressure measurement at every heartbeat using a wrist-mounted device. Omron has developed a multi-element Micro Electro Mechanical Systems (MEMS) pressure sensor integrated with an Application Specific Integrated Circuit (ASIC), resulting in a prototype with 46 pressure sensors designed for continuous blood monitoring [9].

A method for estimating blood pressure by detecting pressure fluctuations from capacitance changes using the tonometry principle has also been developed [10]. This innovative approach involves a wrist- or foot-worn device that houses an array of capacitive sensors, which, when in contact with the skin, can measure the minute displacements caused by arterial pulsations.

### 2.2. Photoelectric Pulse Wave Method

**Figure 2:**
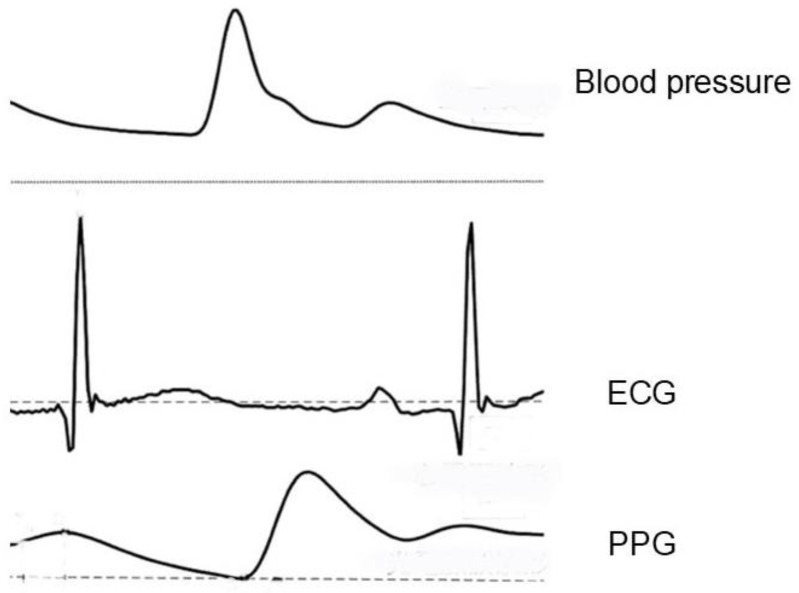
Blood pressure, ECG, and PPG signals.

As depicted in Figure 2, blood pressure and photoelectric pulse waveforms are illustrated, showcasing how blood flow, propelled by the heart’s contractions, is optically detected to generate pulse waves

The PPG signal was obtained through the photoelectric pulse wave method, which is a combination of a light-emitting diode and a photodetector, and research is progressing rapidly because waveforms are easy to obtain. In the photoelectric pulse wave method, green or infrared light is irradiated from the body surface and changes in blood flow, which varies with the pulsation of the heart, are measured as the amount of change in light transmitted through the body.

The method is executed in two primary ways: one involves measuring transmitted light through the fingertips and earlobes, while the other relies on reflected light from the arms. The magnitude of the signal depends on blood volume. The PPG waveform contains the difference between systolic and diastolic blood volume. The typical obtained signal is shown at the bottom of Figure 2. For a detailed PPG theory and techniques, see the reference [11].

From these detected waves, several blood pressure estimation methodologies have been developed, including Pulse Transit Time (PTT), Pulse Contour Method (PCM), and the Acceleration Pulse Wave Method. PTT measures the time delay between the R-wave of an electrocardiogram (ECG) and the corresponding pulse wave arrival at a distant artery, offering insights into arterial stiffness and blood pressure changes. PCM analyzes the contour of the pulse waveform to estimate blood pressure by correlating waveform features with pressure values. The Acceleration Pulse Wave Method assesses the acceleration of the pulse wave along the arterial tree to derive blood pressure data. These methods harness the photoelectric pulse wave’s potential for blood pressure monitoring, offering a promising avenue for non-invasive cardiovascular health assessment.

#### 2.2.1. Pulse Transit Time

**Figure 3:**
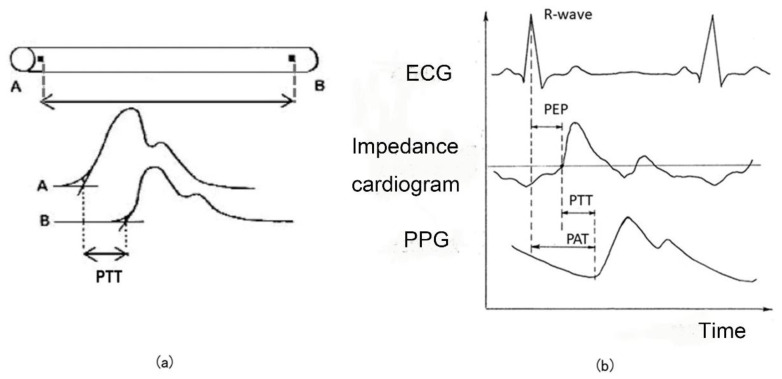
(**a**) Pulse Transit Time (PTT) A and B are the measurement points of PPG and their waveforms are shown. (**b**) Pulse Arrival Time (PAT) with R wave of ECG, Pre-ejection period (PEP), and PTT.

As shown in Figure 3, pulse wave transit time (PTT) can be measured (a) from the pulse wave signals between two points or (b) from the R waves of ECG and pulse wave signals [12,13,14,15]. Strictly speaking, the time between the ECG R wave and the appearance of the pulse wave signal is defined by as the pulse arrival time, or PAT. In other words, PAT = PEP + PTT, which includes the pre-ejection period (PEP) when the arterial valve opens during systole and blood flows into the artery. Despite this distinction, numerous studies have utilized PAT as a surrogate for PTT in blood pressure estimation. The estimation of blood pressure from these timings is often facilitated by employing the Moens–Korteweg equation, which relates arterial stiffness and pulse wave velocity to blood pressure.

#### 2.2.2. Pulse Contour Method

The Pulse Contour Method (PCM) is a hemodynamic analysis technique used to estimate cardiovascular parameters, such as cardiac output, from the dynamics of blood pressure waveform. Central to PCM is the application of Windkessel parameters, which encompass the characteristic impedance, compliance, and peripheral resistance of the aorta. These parameters are instrumental in constructing a linear or non-linear hemodynamic model of the aorta.

The Windkessel model represents the hemodynamics (flow and pressure) in arteries. The flow rate $Q\left(t\right)$ and blood pressure $P\left(t\right)$ in the artery are derived from the peripheral vascular resistance R and the elasticity (compliance) C of the artery. The relationship is expressed as: $Q=C\frac{dp}{dt}+\frac{P}{R}$. This equation delineates the interplay between the rate of pressure change and flow, moderated by the arterial compliance, and the pressure itself, influenced by peripheral resistance. In this model, the behavior of blood flow in arteries is conceptualized as a first-order delay system with flow as input and pressure as output. The pressure waveform is time-delayed with respect to the flow rate. This is referred to as the mechanism by which arterial blood does not stagnate and maintains flow even during the diastole of the left ventricle.

**Figure 4:**
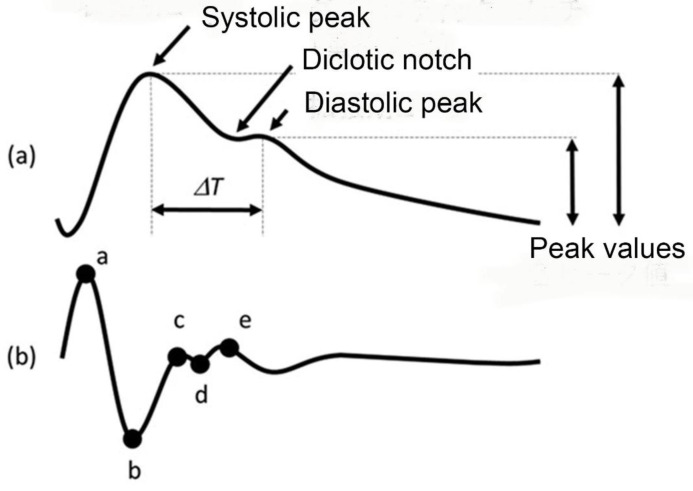
(**a**) PPG pulse contour analysis and Second derivative of PPG signal. (**b**) Its second derivatives. Symbols a–e are the peak points.

This involves applying the hemodynamic model to pulse waves, taking into account reflections from the periphery of the pulse waveform, and estimating blood pressure from the pulse waveform based on the magnitude of the peaks and their time intervals [16,17,18,19]. The pulse wave contouring method is a method of estimating the pressure of the left ventricle during the diastolic phase As illustrated in Figure 4a, the parameters of the PCM are crucial for blood pressure estimation. These parameters are derived from a detailed analysis of the pulse wave’s peak values and their corresponding time intervals. The method leverages the relationship between the pressure and flow waves, where the pressure waveform lags behind the flow wave due to the propagation delay in the arterial system.

#### 2.2.3. Acceleration Pulse Wave Method

Acceleration pulse wave refers to the second derivative of the photoplethysmogram. It was developed to clarify the inflection point of the original finger volume pulse wave and is now also established as an independent test method as an acceleration pulse wave. Currently, as shown in Figure 4b, the components of the accelerated pulse wave are measured as early systolic positive wave (wave a), early systolic negative wave (wave b), mid-systolic rising wave (wave c), late systolic falling wave (wave d) and early diastolic positive wave (wave e).

Blood pressure estimation for this method typically involves multiple regression analysis or machine learning algorithms applied to the original pulse waveform, the second derivative waveform, and associated timing data [20]. In practice, machine learning models often estimate blood pressure by extracting features from the pulse waveform or the acceleration pulse wave components. These features are derived through the pulse wave contour method and the acceleration pulse wave method. For an in-depth understanding of the principles and considerations in these methods, one is advised to consult the relevant literature [5,6].

Detailed analysis of specific methods showed that PCM performed better than PTT for both SBP and DBP, although these differences were not statistically significant [21].

#### 2.2.4. Signal Processing to Estimate Blood Pressure

The characteristics of PPG signals, especially their sensitivity to noise caused by motion artifacts, present a significant challenge in achieving cuffless blood pressure measurement accuracy. Wavelet Transform, which is widely used to remove noise by decomposing signals into different frequency bands, is particularly effective for motion artifacts [22,23]. As the discrete version, Discrete Wavelet Transform (DWT) has been utilized in conjunction with ECG and PPG signals for motion artifact removal and denoising. This step ensures the extracted features are robust to noise, improving estimation accuracy [22,24,25].

Early studies on cuffless blood pressure estimation primarily relied on traditional machine learning models. Linear Regression and Least Squares Regression were among the first to explore relationships between extracted PPG features and blood pressure values [15,26]. Thereafter, more advanced models such as SVM and Random Forest introduced non-linear decision boundaries, achieving acceptable accuracy under resting conditions [27,28].

Given the high redundancy and susceptibility to motion artifacts of raw PPG signals, Feature Extraction Techniques are pervasively used in PPG signal processing. Features such as Pulse Transit Time (PTT), heart rate variability, and PPG derivatives have been critical in these models [27,28]. Despite their robustness, these metrics often struggled with dynamic conditions like exercise or motion.

The past decade has seen significant advances in the application of deep learning models. Convolutional Neural Networks (CNNs) have been used to automatically extract spatial features from PPG and ECG signals, providing robust performance across varying conditions [22,23]. Recurrent Neural Networks (RNNs), such as Long Short-Term Memory (LSTM) Networks, capture temporal dependencies in physiological signals, making them particularly suited for time-series data like PPG [22,24,29]. Generative Adversarial Networks (GANs) have been explored for generating synthetic training data and enhancing the robustness of cuffless BP estimation models [24] or to extract the relevant features used in downstream classification/regression tasks. These techniques surpass traditional methods in capturing the complex non-linear relationships between PPG signals and blood pressure, particularly when combined with physiological parameters like age, gender, height, weight, and BMI [26,29].

While existing methods demonstrate promising results, challenges remain. Motion artifacts and environmental noise continue to impact signal quality, particularly in ambulatory settings. The generalizability of models across diverse populations is limited by variations in physiological characteristics. The lack of standardized datasets and benchmarking protocols hinders direct comparison between methods. Future research should focus on developing robust preprocessing pipelines, incorporating multi-modal sensing technologies, and exploring transfer learning to address population variability.

### 2.3. Ultrasound Method

Ultrasound can create images based on the propagation characteristics of each tissue in the human body and can be used to help diagnose diseases. Blood pressure can also be measured using ultrasound based on the pulse-echo method [28,30,31,32,33,34]. The ultrasound array detects the position of the radial artery and records the diameter waveform, and then the blood pressure is estimated from the obtained accurate blood pressure waveform.

### 2.4. Other Principles Under Research

Other methods include a smartphone-type method where the fingertip is placed in contact with a camera to capture a photoplethysmographic pulse wave of the fingertip and display the blood pressure using a built-in blood pressure estimation program [35], the bio-impedance method [36,37,38], and seismocardiogram [39,40] bio-impedance sensor array built in a flexible wristband or in a ring, with the morphology of arterial pulsatile PTT detection being extracted from each beat of the BI waveforms at various distances. The seismocardiogram (SCG) measures the chest motion caused by cardiac activity, and blood pressure is estimated combined with SCG and PTT. These have only been evaluated at the laboratory level.

## 3. Commercialization of Cuffless Blood Pressure Monitors

The number of research papers published and patents granted for cuffless sphygmomanometers has increased over the past few years, with 951 papers reported in PubMed up to November 2024. This section presents devices that have been approved in the USA, EU and Korea, as shown in Table 1, Table 2 and Table 3.

**Figure 5:**
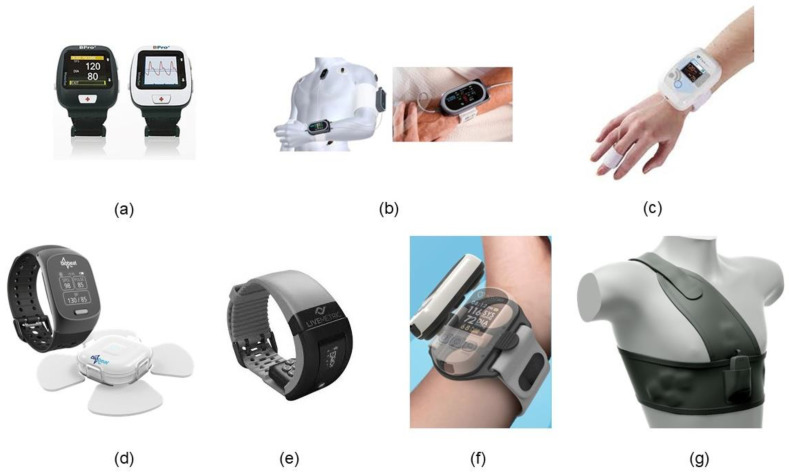
FDA approved cuffless sphygmomanometer (**a**) BPro, (**b**) ViSi Mobile sensor, (**c**) Vital stream, (**d**) Biobeat, (**e**) LiveOne, (**f**) Accurate 24, (**g**) Simple Sence BP [55].

Table 1 shows cuffless sphygmomanometers that have received 510(k) clearance from the US Food and Drug Administration (FDA). Table 2 and Table 3 show the popular cuffless blood pressure monitors obtained from the European Commission CE Marking (CE) and Korea’s Ministry of Food and Drug Safety (MFDS)-approved devices, respectively. These devices are considered approved because of their accuracy in the standard for cuff sphygmomanometers (ISO 81060-2:2018) [41]. The approved devices are classified as those based on the photoplethysmograph or tonometry method. These have been tested in comparison with either auscultatory sphygmomanometers or direct blood pressure measurement methods using catheters in operating theaters and intensive care units to verify the accuracy and validity of cuffless blood pressure monitors. Accuracy, principle, and test procedures are obtained from references [42,43,44,45,46,47,48,49,50,51,52,53,54,55,56,57,58,59,60,61,62]. Of the devices listed in Table 1a, a photograph of the cuffless sphygmomanometer is shown in Figure 5.

**Table 1:** (**a**) FDA-approved devices and (**b**) advantages and disadvantages of FDA-approved devices.

(a)				
Device	Principle	Manufacture	Calibration	FDA 510(k)
BPro [42,43]	Applanation tonometry	HealthSTATS International Pte Ltd. London, UK	Cuff-based	K060315 2006 K131916 2014 K173028 2018
ViSi Mobile Sensor	PAT based on ECG-r PPG	Sotera Digital Health Carlsbad CA. USA	Oscillometric	K112478 2012
Caretaker Vital Stream [16,44,45,46]	PCM with low pressure load	Caretaker Medical, LLC Charlottesville, VA, USA	Cuff-based. Volume clamp Continuous BP monitor	K151499 2016 K163255 2017 K181196 2018 K211588 2021 K213699 2022
Biobeat [47,48,49,50]	PCM	Biobeat Tikva, Israel	Cuff-based Arterial-lines	K190792 2019
LiveONe [51]	Applanation tonometry	LiveMetric (Medical) S.A. New York, NY, USA	Arterial lines	K201302 2022
Accurate 24 Non-invasive blood pressure monitor [52,53,54]	PTT with image sensor	Accurate Meditech Inc New Taipei City, Taiwan	Cuff-based	K222658 2023
SimpleSense-BP, SimpleSense-BP Software Application version 1 [55]	PAT Smart shirt	Nanowear New York, NY, USA	Cuff-based	K232053 2023
(**b**)				
	**Advantage**	**Disadvantage**		**Intended Use**
BPro	Simple use	Difficult to find the exact blood vessel as well as to move a vessel		Home
ViSi Mobile Sensor	Accurate with calibrations before sleeping and after awakening	Stand-alone Mainly used in the operating theater and ICU		Operating room ICU
Caretaker Vital Stream	Highly resistant to noise	Less comfortable		Hospital ward ICU
Biobeat	Watch type: Simply handle Patch type Small and lightweight, suitable for long-term monitoring	Motion artifact Patch type is reliable		Watch type Home Patch type Operating room
LiveONe	Simple use	Difficult to find the exact blood vessel as well as to move a vessel		Home
Accurate 24 Non-invasive blood pressure monitor	Unaffected by external environmental factors, high accuracy	Larger sensor size, less portable		Hospital ward
SimpleSense-BP Software Application	Simple structure, suitable for measuring curved areas	Needs tight contact Susceptible to interference from sweat and other external factors		Home Sport

**Table 2:** Typical cuffless blood pressure monitor with European CE marked.

Device	Principle	Manufacture	Calibration
Freescan BPM-490 [56]	PPG + ECG	Maisense, Spring Lane, TX, USA	Auscultation
SOMNOtouch NIBP [57]	PPG + ECG	Somno Medics Randersacker, Germany	Auscultation
Aktiia [58,59,60]	PCM	Aktiia SA Neuchâtel, Switzerland	Auscultation Arterial line

**Table 3:** Korea’s MFDS approved devices.

Device	Principle	Manufacture	Calibration
Galaxy A [61]	PCM	Samsung Electronics Seoul, South Korea	Arterial line
CART-1 Plus [62]	PCM	Sky Labs, Seongnam, South Korea	24 h ABPM

The FDA approved several cuffless blood pressure monitors with PAT, PCM, and tonometry. The first device approved was a tonometry-based device. Tonometry measures blood pressure directly from the radial artery, but finding the right location is extremely difficult. Typically, a sensor array is placed on the arm and the high pressure value of the sensor array is detected as blood pressure. Accurate 24 uses ultrasound images to find the correct location of the radial artery. The ViSi sensor is used as an overnight blood pressure monitor. Before and after monitoring, the signals obtained are calibrated by the cuff-based sphygmomanometer. The interpretation techniques provide relatively high accuracy.

Caretaker current Vital stream contains a finger sensor and interface. The finger sensor is placed with 2–3 mmHg pressure to prevent the motion artifact and measure the finger blood pressure.

The first watch-type device is Biobeat. At the same time, the company produces the patch type. The watch type is weak to motion artifact and it is difficult to monitor in the long term. A smart shirt type is also produced. ECG and PPG are measured, and the shirt must be tight-fitting for correct monitoring.

The advantages and disadvantages of the FDA approved devices are shown in Table 1b.

Cuffless blood pressure monitors can be divided into those that measure blood pressure continuously on their own (stand-alone), wearable types that measure blood pressure continuously or intermittently, and smartphone types that measure blood pressure intermittently. Stand-alone types are small, portable, and can be used for emergency blood pressure measurement [56,57]. Wearable sensors transmit signals acquired from a smartwatch or wristband to a smartphone, touch screen, or PC wirelessly for daily blood pressure management and maintaining health. Many of them have both an activity tracker function with a built-in accelerometer and a blood pressure measurement function based on pulse wave propagation time [42,43,44,45,46,47,48,49,50,51,52,53,54,55,58,59,60,61,62]. Recently, the easy availability of ECG modules and photoelectric pulse wave modules has made it possible to easily measure blood pressure using the photoelectric pulse wave method, and many models without medical approval can be found in the market.

The goal of the development of a device that measures blood pressure without a cuff is to satisfy the resting accuracy of 5 ± 8 mmHg of conventional cuff-type non-invasive blood pressure monitors. Stability and adequacy for long-term use are important but have not been standardized

## 4. Clinical Applications

The dissemination of inexpensive, unbreakable, and simple blood pressure monitors is necessary to prevent the increase in the number of hypertensive patients, especially in low- and middle-income countries, and the rise in cardiovascular disease. It could also be used as a blood pressure monitor for screening purposes in health check-ups and medical examinations.

The validity of long-term measurement has been compared with a commercially available ambulatory blood pressure monitor (ABPM) for 24 h [59], nocturnal blood pressure variation [39], and the validity of one-month use has been confirmed. Secondly, continuous blood pressure measurement in the operating theatre has been reported [60]. This was done with catheters in the arteries and simultaneous measurements showed a high correlation.

On the other hand, another group reported inferior accuracy in comparative measurements with ABPM [63] and in intensive care units [64]. Henry et al. [65] summarized further improvements of cuffless sphygmomanometers in clinical practice. They cite the motion artifact nature of the PPG signal and suggest that the design of the PPG sensor should be optimized to reduce the effects of motion artifacts. In addition, because body position can affect the accuracy of blood pressure measurement, cuffless monitors are often worn on the wrist, and validation protocols need to assess how changes in the elevation of the arm relative to the heart may affect the accuracy of blood pressure measurement [66]. They also suggest that the lack of standardized accuracy validation hinders the use of cuffless blood pressure monitors in clinical practice.

There have been requests from physicians to promote the use of cuffless sphygmomanometers, for which the European Society of Hypertension has issued a consensus position [67,68,69]. Although cuffless sphygmomanometers are a promising technology for the management of hypertension, they use different principles, such as the photoelectric pulse wave method and tonometry, which require calibration with cuff sphygmomanometers, and have different purposes, such as screening and home BP measurement. Therefore, standardization of accuracy, tracking of dynamic variations and suitability for long-term use is required. For widespread use in clinical practice, evidence is needed not only for measurement in healthy subjects, but also in pre-hypertensive and hypertensive patients, to ensure that the system can be used in the same way as, or instead of, current blood pressure monitors. Therefore, until a device is developed that can meet these requirements, its use in clinical practice cannot be recommended.

The statement of the European Society of Hypertension Working Group in 2022 is summarized below.

Cuffless sphygmomanometers are already on the market, some of which claim to provide accurate measurements. These devices are heterogeneous in measurement principle, intended use, function, and configuration, and have specific accuracy issues that require different validation from conventional cuff sphygmomanometers. To date, there is no generally accepted protocol for sphygmomanometer validation methods to ensure adequate accuracy for clinical use. Six validation tests have been defined as the most commonly used and recommended as validation procedures for intermittent cuffless sphygmomanometers, with comparative measurements at 30-to-60-min intervals, or at the start of the user, at intervals of 30 s or longer: Static test (absolute blood pressure accuracy); Sphygmomanometer Position Test (robustness to hydrostatic effects); Treatment Test (blood pressure lowering accuracy); Wake/Sleep Test (blood pressure change accuracy); Exercise Test; and Recalibration Test (calibration stability over time). Not all of these tests are required for a particular blood pressure monitor. The validation required will depend on whether the device requires individual user calibration, whether automatic or manual measurements are taken, and whether measurements are taken in multiple positions.

In conclusion, the validation of cuffless sphygmomanometers is complex and needs to be tailored according to their function and calibration. These European Society of Hypertension (ESH) recommendations provide specific, clinically meaningful, and practical validation procedures for different types of intermittent cuffless sphygmomanometers, ensuring that only accurate sphygmomanometers are used for the assessment and management of hypertension [67].

To maintain the reliability of blood pressure monitors for clinical use, two medical device assessment bodies have been identified: the Dabl Education Trust [70] and the STRIDE BP international initiative for accurate blood pressure measurement [71]. These publish recommendations for blood pressure monitors on the web based on evidence papers from validation trials based on accuracy verification according to protocols such as the Association for the Advancement of Medical Instrumentation (AAMI), ESH, and the British Hypertension Society (BHS). The reports are published on the Internet but cuffless sphygmomanometers are not mentioned in these reports [69].

## 5. International Standardization and Approval of Cuffless Sphygmomanometer as Medical Devices

Standards and standardization differ depending on whether cuffless sphygmomanometers are considered an extension of cuff-based sphygmomanometers or treated as a continuous blood pressure monitor. The ESH considers them to be completely different devices. With the spread of AI, there are proposals for cuffless sphygmomanometers that do not require calibration, depending on the algorithm. In resting conditions, calibration may not be required using the AI algorithm, or calibration with a conventional cuff sphygmomanometer may be required. When used as a continuous blood pressure monitor, how does it track dynamic blood pressure fluctuations, and what kind of validation experiments are used to verify its accuracy? In the past and present, evaluations have followed cuff-based sphygmomanometer evaluation protocols such as those of the Association for the Advancement of Medical Instrumentation (AAMI) and ESH (now aligned with the International Standards Organization ISO), but as stated in the ESH recommendations, there is a need for new standards. The Institute of Electrical and Electronic Engineers (IEEE) has been active in standardizing cuffless blood pressure monitors, publishing the IEEE1708 Standard for Wearable Cuffless Blood Pressure Measuring Devices in 2014 [72]. It was based on the ISO 81060-2:2018 standard for cuff blood pressure monitors, but it was pointed out that the number of samples for accuracy evaluation was small, and that it was necessary to add considerations for noise control and posture during calibration. IEEE 1708a-2019 (Amendment 1), which corrected this, was published in October 2019 [73]. The IEEE standard is a standard for wearable devices such as home blood pressure monitors and not necessarily a standard adapted to continuous measurement. Activities to create a standard for continuous sphygmomanometers are ongoing. Meanwhile, the ISO standard for beat-to-beat continuous blood pressure measurement mainly uses the volume compensation method and was published as ISO 81060-3:2022 Non-invasive blood pressure monitoring part 3. However, this does not include cuffless BP measurement, which is being discussed by the ISO Working Group on Non-invasive BP Measurement (ISOTC 121/SC3/JWG7) [74]. The cuffless sphygmomanometers should be a separate standard as their main scope of application is for home use and continuous morning and evening use, whereas ISO 81060-3:2022 is different in being used for intraoperative, emergency care, and ICU. Since then, efforts to create a standard have continued, partly due to the increased clinical use. A standard for cuffless blood pressure needs to be developed as soon as possible.

Next, the initiatives of medical device certification bodies are presented. There are six cuffless blood pressure monitors approved by the US Food and Drug Administration (FDA), as shown in Table 1. All are Class II approved; in March 2012, the ViSi Mobile system (Sotera Digital Health, Carlsbad, CA, USA) was the first to receive Class II certification in combination with a PPG and cuff system. The principle is to monitor nocturnal blood pressure changes using ECG R-waves and photoelectric pulse waveforms. It must be calibrated with a cuff blood pressure monitor before going to sleep and after waking up. Caretaker (Caretaker Medical LLC, Charlottesville, VA, USA), later Vital stream, was approved in October 2018. The principle is to estimate blood pressure using a pulse wave contour method. A stable signal is obtained by squeezing the finger with a fingertip cuff at low pressure (30–45 mmHg). In addition, a wristwatch type BB-613 (Biobeat Technologies LTD, Tikva, Israel) was approved in August 2019, but uses LEDs with multiple wavelengths (presumably estimated using the pulse contour method, but details of the device are not disclosed). The Accurate 24 non-invasive blood pressure monitor, which uses pulse wave propagation time with strict positioning of the radial artery, was approved in 2023. Another device using the applanation tonometry, the BPro (HealthSTATS Technologies London, UK), was approved in 2018, and the LiveOne integrated into a wristwatch in 2022. In addition, SimpleSense-BP will be approved to be embedded into smart shirts in 2023.

As mentioned above, these certification standards apply to cuffed sphygmomanometers, which are mainly used at rest. The characteristic feature of cuffless sphygmomanometers is continuous measurement, for which there is an urgent need for standards.

## 6. Future Consideration

The advantage of cuffless sphygmomanometers is that they allow continuous blood pressure measurement. The challenge is not only to assess blood pressure at rest but also to correctly track blood pressure values during dynamic fluctuations. Another issue is the validity of the system for long-term use. Originally, motion artifact is a major problem in optical measurement with PPG and tonometry. Also, since blood pressure is estimated from volume, it is not a linear relationship. In addition, when the blood vessels are soft, they are highly dilated (normotensive). On the other hand, if the blood vessel is stiff, it requires more force to dilate and also dilates less. Physiologically, it is difficult to solve this model with appropriate parameters. Because of these characteristics, ESH proposes a calibration method for several environments, but for home use it is time-consuming even for the manufacturer to validate.

In the hospital setting, the validity of the EHS proposal should be considered. In the home setting [67,75], the resting blood pressure accuracy of 5 ± 8 mmHg should be met, and dynamic blood pressure should be measured with the Valsalva maneuver test for blood pressure to decrease and the cold stress test for blood pressure to increase. For the validity of long-term measurement, a validation study performed once a day for about one month comparing the value with a reference value from a medically approved automatic blood pressure monitor should be sufficient. In short, it needs to be approved as a medical device, but in terms of safety issues, if the participants detect irregular and abnormal blood pressure values, they will notify the family physician,

It will be important for the widespread use of cuffless sphygmomanometers to establish a standard that is accurate and valid but can be used as an off-the-shelf or over-the-counter device. Clearly stating the purpose of use and performing validation in accordance with that purpose may lead to the widespread use of cuffless sphygmomanometers with greater reliability.

## 7. Conclusions

The current state of the development of cuffless sphygmomanometers is reported. Many devices have been commercialized, leveraging their ease of use and patient-friendly design. Despite their market presence, several challenges must be surmounted to address underlying principles and clinical skepticism. The clinical utility of these devices, particularly in the context of nocturnal blood pressure variability and their performance under antihypertensive treatment, remains a subject of debate It is imperative that cuffless sphygmomanometers demonstrate accuracy, validity, and reliability in clinical settings to gain acceptance for broader medical application. Once accuracy and validity have been demonstrated, it would be important to put the device on the market and leave the decision on the device to the medical community. There have been cases where ear thermometers, fingertip and wrist sphygmomanometers have been medically approved and placed on the market, but have not been used due to inaccuracies in clinical practice, and therefore it is necessary to leave the evaluation to clinicians. The market introduction of such devices should be complemented by ongoing clinical evaluation to ensure they meet the stringent standards of medical practice. The development and certification of new technologies are anticipated with keen interest, as they hold the promise of expanding research horizons and transforming our approach to cardiovascular health.

Cuffless blood pressure measurement offers new possibilities for non-invasive, continuous and dynamic monitoring, but more sophisticated devices are expected to enter the market. This has the potential to redefine our understanding of blood pressure and hypertension.

## Data Availability

Data sharing not applicable.
